# INFα-2b inhibitory effects on CD4^+^CD25^+^FOXP3^+^ regulatory T cells in the tumor microenvironment of C57BL/6 J mice with melanoma xenografts

**DOI:** 10.1186/s12885-016-2473-0

**Published:** 2016-07-07

**Authors:** Yang Yu, Run Huang, Xiangyun Zong, Xiangming He, Wenju Mo

**Affiliations:** Department of Breast Surgery, Zhejiang Cancer Hospital, 38 Guangji Road, Hangzhou, 310022 China; Department of Breast Surgery, Shanghai Jiao Tong University Affiliated Sixth People’s Hospital, 600 Yishan Road, Shanghai, 200233 China

**Keywords:** Melanoma, Tumor microenvironment, CD4+CD25+Foxp3+ Treg_s_, Chemotherapy, Immunotherapy

## Abstract

**Background:**

Regulatory T cells (Treg_s_), particularly the CD4^+^CD25^+^Foxp3^+^ Treg_s_, down regulate immunity and promote tumor cell growth by directly suppressing CD8^+^ and CD4^+^ T cells. Alternatively they can promote tumor growth by generating interleukin-10 (IL-10) and transforming growth factor β (TGFβ) in situ, which help tumor cells to evade the immune system.

**Methods:**

In vivo tumor models were prepared via subcutaneous injection with a suspension of B16 melanoma cells into the left upper flank of C57BL/6 J mice. The mice were randomized into five groups: radiotherapy (RT), chemotherapy (CT), radiochemotherapy (RCT), Inteferon α (INFα) groups, and a control group. Flow cytometry was used to determine the Treg_s_ levels in the spleen and peripheral blood, and immunohistochemistry was performed to determine the expression levels of TGFβ and IL-10 in the tumor microenvironment.

**Results:**

Tumor weight was significantly reduced in the CT or RCT groups (40.91 % and 41.83 %, respectively), while the reduction in tumor weight was relatively lower for the RT and IFNα groups (15.10 % and 13.15 %, respectively). The flow cytometry results showed that the ratios of CD4^+^CD25^+^Foxp3^+^ Treg_s_ to lymphocytes and CD4^+^ cells in the spleen and in peripheral blood were significantly decreased after treatment with IFNα (*P* < 0.05). Expression of TGFβ and IL-10 in the tumor microenvironment in the CT and RT groups was higher compared with the control group (*P* < 0.01), while the expression of TGFβ and IL-10 in the INFα group was not significantly different (*P* > 0.05).

**Conclusions:**

The results show that INFα-2b inhibits cancer cell immune evasion by decreasing the levels of CD4^+^CD25^+^Foxp3^+^ Treg_s_ and suppressing the expression of TGFβ and IL-10 in the tumor microenvironment.

## Background

Although melanoma is not a common tumor worldwide [1], it is the most lethal form of all skin cancers. In recent years, its morbidity has increased more than any other solid tumor. In 2012, 232,000 new melanoma cases were reported throughout the world, accounting for 2 % of all tumors. It was reported that there were 76,100 new melanoma cases, and 9719 deaths, in the USA in 2014, accounting for 4.6 % and 1.7 % of all tumors, respectively [2]. Other than classical therapies, such as surgery, radiotherapy and chemotherapy, immunotherapy is playing an important role in the treatment of melanoma. However, melanomas have been found to evade the immune system, thus making them a refractory type of cancer.

Regulatory T cells (Treg_s_), particularly CD4^+^CD25^+^Foxp3^+^ Treg_s_, down regulate immunity and promote tumor cell growth [3]. Treg_s_ can be recruited to the microenvironment of melanoma because of the chemotaxis of chemokine (C-C Motif) Ligand 22 (CCL22) that is produced by tumor cells [4]. As a result, infiltrated Treg_s_ can exert suppressive effects on effector CD8^+^ and CD4^+^ T cells either through direct cell-to-cell contact [5, 6] or indirectly by generating interleukin-10 (IL-10) and transforming growth factor β (TGFβ) in situ [7–9], which helps tumor cells to evade the immune system. Moreover, it has been reported that Treg_s_ can be directly induced by melanoma cells, thus further suppressing the immune system in the tumor microenvironment [10, 11].

TGFβ, which is a secreting protein that modulates cell proliferation and differentiation, has dual effects in tumor initiation and progression. In the early stage of tumorigenesis, TGFβ is a tumor suppressor, whereas in advanced tumors, TGFβ promotes tumor angiogenesis, invasion, metastasis, and immunosuppression [12]. Interleukin-10, also known as human cytokine synthesis inhibitory factor, is an anti-inflammatory cytokine which plays a critical role in preventing an immune response and autoimmune pathologies [13]. Nevertheless, in tumorgenesis, IL-10 inhibits the expression of antigen presenting cells (APCs) and further prevents the dendritic cell (DC)-mediated transformation of T cells into cytotoxic T cells (CTLs). It also affects CD8^+^ T cells, which further promotes tumor initiation and progression [14]. Both TGFβ and IL-10 in the tumor microenvironment can be excreted by Treg_s_ and tumor cells, thus mediating the immunosuppressive effect of Treg_s_ [9]. As such, TGFβ and IL-10 can be regarded as two significant immunosuppressors in the melanoma microenvironment. However, the role of these two cytokines in the tumor microenvironment still remains elusive when challenged to differential clinical therapies.

As different treatment strategies have different effects on the tumor microenvironment, it is of interest to investigate their effects and relevant mechanisms. As such, in this study C57BL/6 J mice bearing melanoma were used as tumor models and treated with either radiotherapy (RT), chemotherapy (CT), radiochemotherapy (RCT), or intravenously administered Inteferon α-2b (IFNα-2b). After treatment, the level of Treg_s_ in the spleen and in peripheral blood, and the levels of TGFβ and IL-10 in the tumor microenvironment were determined.

## Methods

### Mice model construction and group intervention

Thirty female C57BL/6 J mice were purchased from Shanghai Slack laboratory Animal Co. Ltd (Shanghai, China). Mice were 6-weeks old at the start of the experiments, weighing 20 ± 2 g. B16 melanoma cells were harvested in their logarithmic growth phase and were made into a single cell suspension (2.5 × 10^7^ cells/mL). Each mouse was then subcutaneously injected with 0.2 mL of the cell suspension (about 5 × 10^6^ cells) in their left upper flank.

Thirty successfully B16 melanoma inoculated C57BL/6 J mice were randomly divided into five groups: RT, CT, RCT, INFα groups, and a control group, with six mice per group. For each group, treatment was started 7 days after inoculation. Mice in the RT group were given a single conformal treatment of 500 cGy of radiation (source skin distance (SSD) = 100 cm, using a 5 cm × 5 cm module body filled lead-antimony alloy, and 1.5-cm-thick physical tissue equivalent compensation filmed on the surface of the irradiated skin). Tail vein injection and intraperitoneal injection (i.p.) of normal saline (NS, Zhejiang Shapuaisi Pharmaceutical Limited, Pinghu, China) were used as the control intervention for the IFNα and chemotherapy groups, respectively [15, 16]. For the CT group, each animal was intraperitoneally injected with 40 mg/kg dacarbazine (DTIC, Nanjing Pharmaceutical Factory Co, Ltd, China) daily from days 9 to 15, and NS was injected into the tail vein as a control for IFNα. Mice in the IFNα group were administrated with INFα-2b (10,000 U per mouse, Schering-Plough Corporation, USA) via tail vein injection on days 7, 9, 11, 13 and 15, and NS was injected i.p. as an alternative control for DTIC [17]. For the RCT group, the mice were given identical treatments synchronously with the RT and CT groups, with tail vein injection of NS as a control treatment. For the control group of mice, NS was administered via i.p. and tail vein injection simultaneously with the CT and INFα groups.

On day 16, all mice had completed their treatments and were sacrificed. Postocular blood (1 mL) was collected before the animals were euthanized and the samples treated with 3.8 % sodium citrate to prevent coagulation. The spleens were also excised and subcutaneous tumor xenografts were excised completely for further examination.

### Cell suspension preparation, antibody labeling and flow cytometry

C57BL/6 J mice were euthanized by the cervical dislocation method. Their spleens were excised on a clean bench and ground on 200-mesh nylon before the filtrate was collected and centrifuged at 1.5 × 10^3^ rpm for 5 min. The supernatant was discarded and the cells were resuspended in phosphate buffered saline (PBS) for further experiments.

Separating medium (2 mL Percoll, Santa Cruze, Shanghai, China) was added to a centrifuge tube. Previously collected blood (2 mL) was then added to the separating medium, and the samples centrifuged for 15 min at 3 × 10^3^ rpm. The lymphocytes were then separated and transferred to another centrifuge tube and spun for 5 min at 1.5 × 10^3^ rpm, before they were resuspended with PBS for further experiments.

Collected peripheral blood and spleen cell suspensions were centrifuged at 1.5 × 10^3^ rpm in order to collect the cells in a 100 μL flow cytometry staining buffer system. Mouse Regulatory T cell staining kit #1 (eBioscience, San Diego, CA, USA) was used to label the cells as per the manufacturer’s instructions. Anti-mouse CD4 (0.125 μg) and anti-mouse CD25 (0.06 μg) were added to each reacting system and incubated in the dark for 30 min at 4 °C. After surface antibody labeling, the cells were twice washed with flow cytometry staining buffer, before fixation/permeabilization working solution (1 mL) was added to resuspend the cells, before they were incubated overnight in the dark at 4 °C. The cells were then twice washed with permeabilization buffer, before 0.5 μg of Fc blocker (CD16/32) was added, and the cells were incubated again in the dark for 15 min at 4 °C. Finally, anti-mouse/rat Foxp3 antibody (0.5 μg) was added and the cells incubated in the dark for 30 min at 4 °C. The cells were then twice washed with permeabilization buffer before detection, using 500 μL of permeabilization buffer to resuspend the cells. Flow cytometry was undertaken using an Accuri C6 Flow Cytometer (BD Biosciences, San Jose, CA, USA).

Lymphocyte clones were first selected from a FSC-A/SSC-A scatterplot, then CD4^+^ T cells clones were selected through a CD4 lymphocyte clone/SSC–A, and finally CD4^+^CD25^+^Foxp3^+^ Treg cells were distinguished from CD4^+^ T cells by gating CD25/Foxp3 and homotype contrast with Foxp3. The proportion of CD4^+^CD25^+^Foxp3^+^ Treg_s_ to CD4^+^ T cells and lymphocytes was calculated to evaluate the level of CD4^+^CD25^+^Foxp3^+^ Treg_s_ alteration, and the results analyzed using t-tests.

### Tumor tissue immunohistochemistry

Formalin-fixed paraffin sections were prepared and dried overnight at 37 °C. Following dewaxing in xylene and rehydration with alcohols, endogenous peroxidase was inactivated in 3 % H_2_O_2_ at 37 °C for 10 min. Microwave antigen retrieval was completed using a citric acid buffer (0.01 M, pH 6.0, Maixin Biotech, Fuzhou, China) and cooled to room temperature. Immunohistochemistry was performed with primary IL-10 (Abcam, ab34843, Cambridge, UK) and TGFβ antibodies (Abcam, ab66043). Slides were incubated with both primary antibodies at a dilution of 1:100 overnight at 4 °C with PBS used as a negative control, and balanced for 30 min at room temperature. After washing with PBS, the slides were incubated at a 1:200 dilution of goat anti-rabbit secondary antibody (Abcam, GR101082-1) for 60 min at room temperature. They were then washed with PBS, DAB substrate kit (Zhongshanjinqiao Biotech, Beijing, China) and used to develop the slides which were redyed with hematoxylin (Sigma, St. Louis, MO, USA).

Three fields were randomly chosen for microscopic study (×200) for each immunohistochemical slide. Each tissue section was semi-quantitatively scored according to the percentage of positive cells and the staining intensity. We assigned the following proportion scores: 0 if 0–5 % of the tumor cells showed positive staining, 1 if 6–25 % of cells were stained, 2 if 26–50 % were stained, 3 if 51–75 % were stained, and 4 if over 75 % of the cells were stained. We rated the intensity of staining on a scale of 0 to 3: 0, negative; 1, weak; 2, moderate; and 3, strong. We then combined the proportion and intensity scores to obtain a total positive score (range, 0–12): score 0 is negative, a score of 1 to 6 is weakly positive, and a score of 7 to 12 is strongly positive.

### Statistical analysis

All experimental data is presented as mean ± standard deviation, and analysis of variance (ANOVA) was performed to compare the data of different groups using the statistical analysis system 9.3 (SAS, Cary, NC, USA). A *P* value <0.05 was considered as statistically significant.

## Results

### RT, CT and IFNα significantly inhibit melanoma growth

**Fig. 1:**
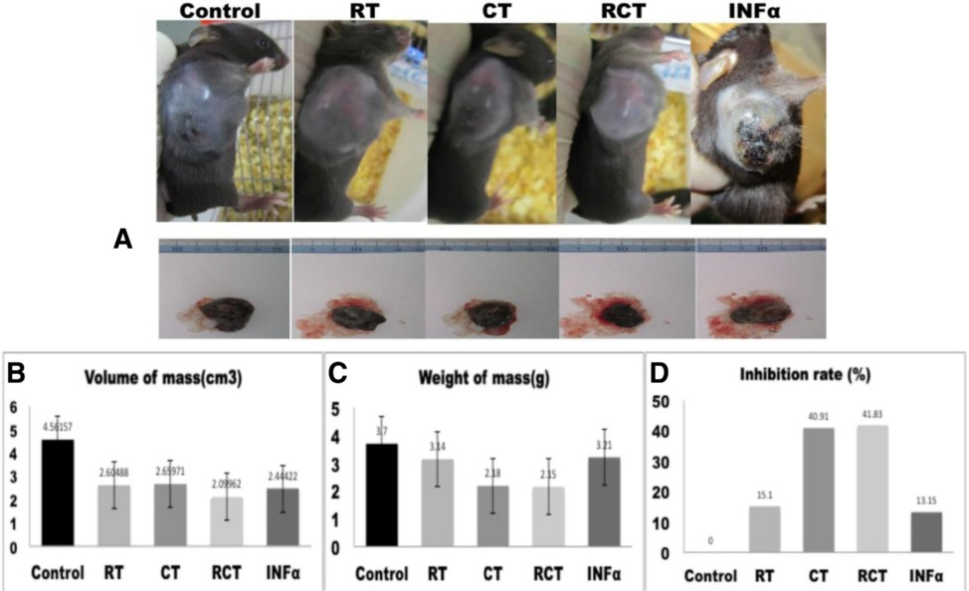
Changes in the tumors after treatment with different therapies. **a** C57BL/6 J mice and excised tumors from each group. **b** Volumes of the tumors treated with radiotherapy (RT), chemotherapy (CT), radiochemotherapy (RCT) and IFNα were significantly smaller than those of the control group, *p* < 0.05. V = 4/3πabc, a. major radius, b. minor radius, c. radius perpendicular to minor axis, measured by ultrasound. **c** Excised tumors were weighed using an analytical balance. Tumor weights were reduced significantly in the CT and RCT groups, while only mildly reduced in the RT and IFNα groups. **d**. Tumor inhibition rate ((W_control_-W_treatment_)/W_control_ × 100 %) after treatment based on the tumor mass

C57BL/6 J mice were inoculated with B16 cells to produce an in vivo xenograft model of melanoma. The mice were divided into five groups (*n* = 6), and treated with a range of different therapies, including: RT, CT, RCT, and IFNα. As shown in Fig. 1b, the tumor volumes of the mice treated with different therapeutic regimens were all smaller when compared with the tumor volumes of the control group mice. This result indicates that tumor growth can be significantly inhibited by conventional RT, CT, RCT, and IFNα. When comparing tumor weights, we calculated the tumor inhibition based on the mean weight of each group (Fig. 1c, 1d). Tumor growth was better suppressed when mice were given CT and RCT (40.91 % and 41.83 %, respectively) when compared with the tumors of the RT and IFNα groups (15.10 % and 13.15 %, respectively), as shown in Table 1.

**Table 1:** Melanoma tumor mass after treatment with different regimens

Groups	Control	Radiotherapy	Chemotherapy	Chemoradio-	INFα
Treatment	None	6 MeV X-ray 500 cGy	DTIC 40 mg/kg,D9-15	X-ray + DTIC	IFNα-2b 10000U D7,9,11,13,15
Number of mice	6	6	6	6	6
Weight of mass (g)	3.70 ± 0.94	3.14 ± 0.90	2.18 ± 0.77	2.15 ± 1.03	3.21 ± 1.86
*p* value		0.366	0.024	0.039	0.617
Volume of mass (cm^3^)	4.56 ± 0.94	2.60 ± 0.68	2.66 ± 1.18	2.10 ± 1.12	2.44 ± 1.37
*p* value		0.005	0.023	0.006	0.021
Inhibition rate (%)	N/A	15.1	40.91	41.83	13.15

### IFNα significantly suppresses CD4^+^CD25^+^Foxp3^+^ Treg_s_ levels

**Fig. 2:**
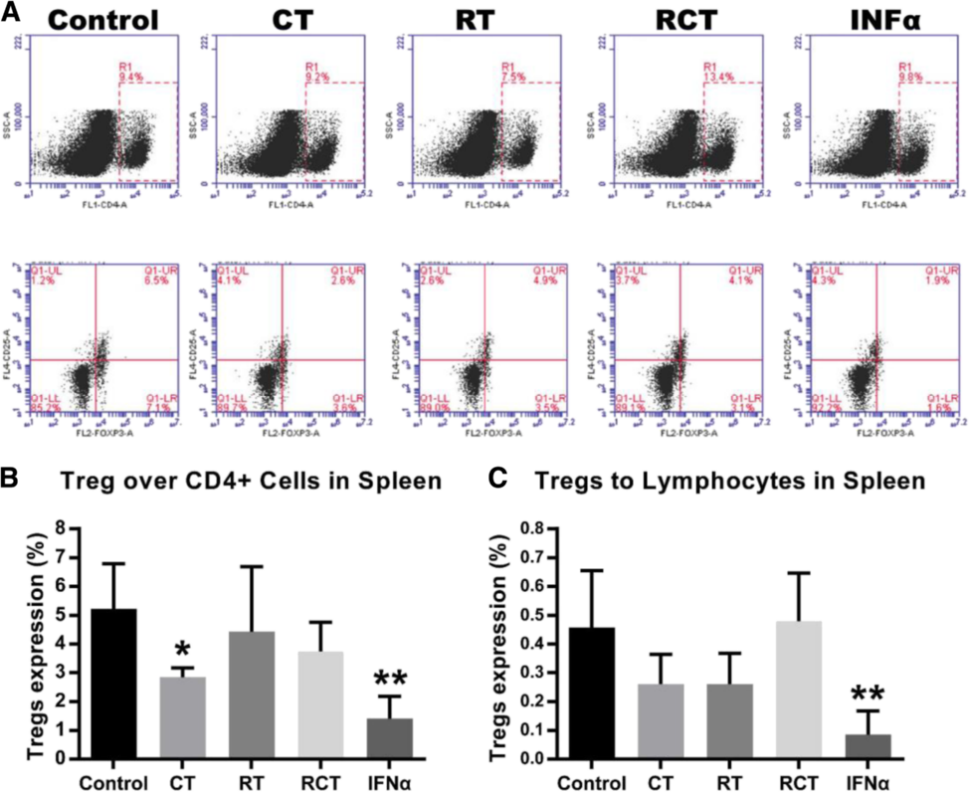
Flow cytometry assay of mice spleen tissue. **a** Gating strategies of CD4^+^ T cells and CD25^+^FOXP3^+^ Treg_s_ for spleen tissue. **b** and **c** Proportion of Treg_s_ to CD4^+^ T cells and lymphocytes in the spleen. * indicates *P* < 0.05 and ** indicates *P* < 0.01 compared with the control group

Flow cytometry was used to determine Treg_s_ levels in the spleen and in peripheral blood of the mice after completion of their treatments. In comparison to the control group, the flow cytometry assay showed that the mouse spleen levels of CD4^+^CD25^+^ Foxp3^+^ Treg_s_ were significantly down regulated in INFα group, compared to the CD4^+^ T cells (*P* < 0.01) and to the lymphocyte cells (*P* < 0.01). The decrease in CD4^+^CD25^+^Foxp3^+^ Treg_s_ levels of the CT group was significant when compared with the CD4^+^ T cells (*P* < 0.05), but not significant when compared with the lymphocytes (*P* > 0.05) (Fig. 2b, 2c).

**Fig. 3:**
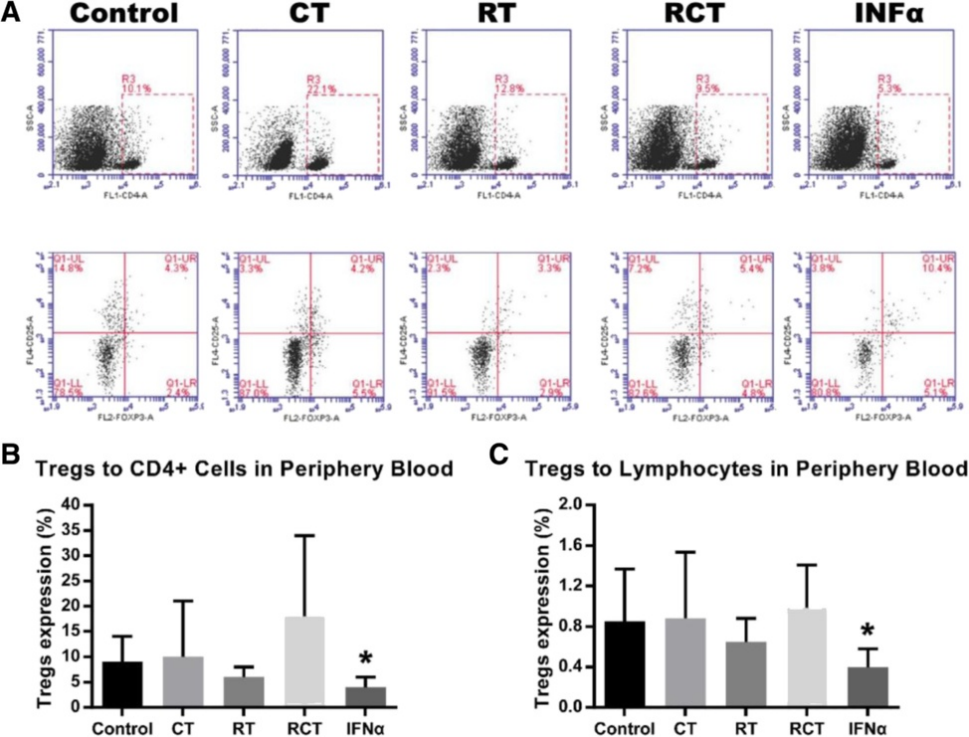
Flow cytometry analysis of mice peripheral blood. **a** Gating strategies of CD4^+^ T cells and CD25^+^FOXP3^+^ Treg_s_ for peripheral blood. **b** and **c** Proportions of Treg_s_ to CD4^+^ T cells and lymphocytes in peripheral blood. * indicates *P* < 0.05 and ** indicates *P* < 0.01 compared with the control group

The results for the peripheral blood samples showed a significant decrease in the IFNα treatment group for both CD4^+^CD25^+^Foxp3^+^ Treg_s_ compared with the CD4^+^ T cells and compared with the lymphocytes (*P* < 0.05), although there was no significant change in the CD4^+^CD25^+^Foxp3^+^ Treg_s_ levels for the RT, CT and RCT groups (Fig. 3). The data indicates that IFNα-based immunotherapy can significantly down regulate the level of CD4^+^CD25^+^Foxp3^+^ Treg_s_ while RT and CT have little effect.

### RT and CT upregulate expression of TGFβ and IL-10 in tumor microenvironment

**Fig. 4:**
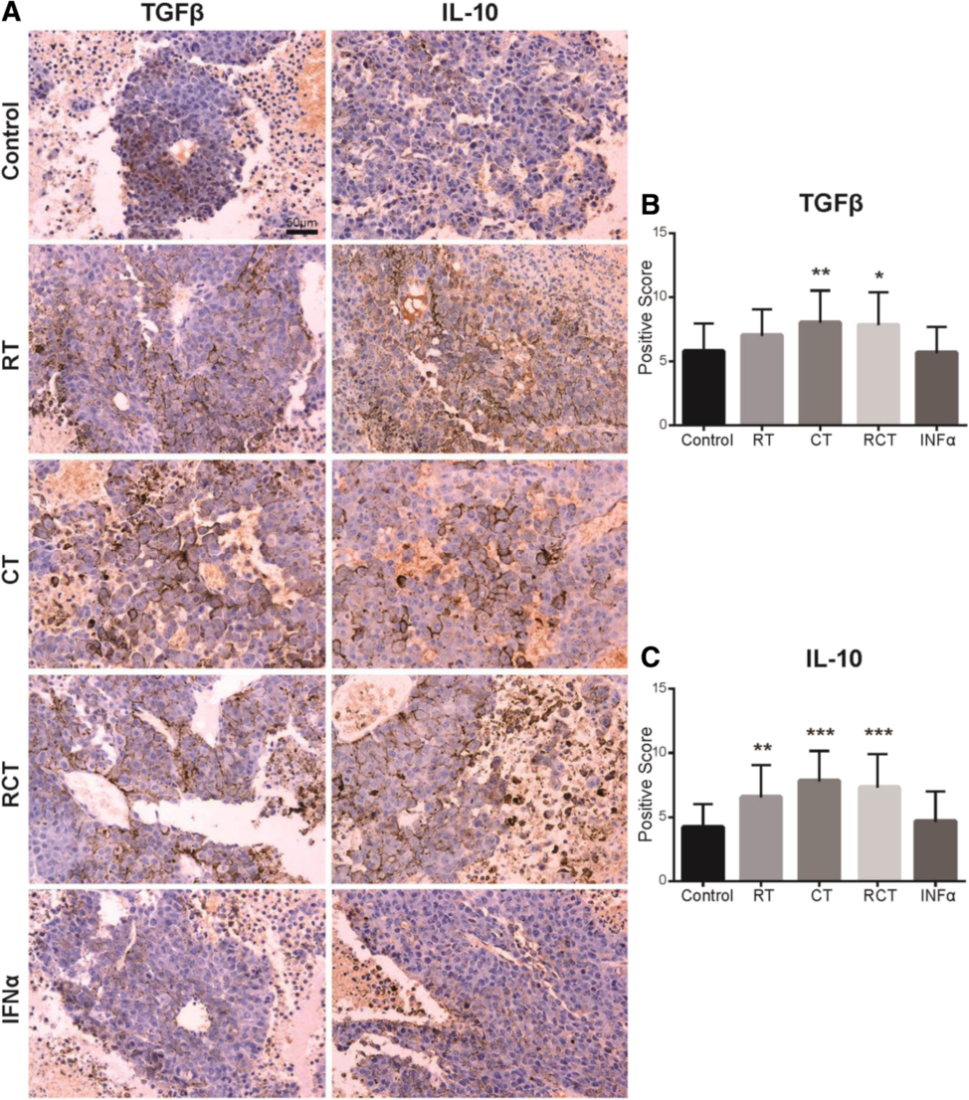
Immunohistochemistry and positive scores of TGFβ and IL-10 in the tumor microenvironment. **a**. Immunohistochemistry graphs of tumor xenografts in C57BL/6 J mice, post treatment. Extensive expression of TGFβ and IL-10 could be detected in the area surrounding the melanoma cells post radiotherapy and chemotherapy, while IFNα had little effect on the expression of TGFβ and IL-10. **b** and **c** Immunohistochemistry positive scores of TGFβ and IL-10. * indicates *P* < 0.05, ** indicates *P* < 0.01 and *** indicates *P* < 0.001 compared with the control group

To investigate the changes in TGFβ and IL-10 levels in the tumor microenvironment, immunohistochemistry was used to examine TGFβ and IL-10 expression in the xenografts. As shown in Fig. 4a, the expression of TGFβ and IL-10 in RT and CT treated mice were much higher when compared to the levels in the tumors of the control group. The results showed that the expression levels in the CT were significantly different to the control group for TGFβ (*p* < 0.01) and IL-10 (*p* < 0.001). In addition, RT was found to increase the expression of IL-10 in the surrounding cancer cells (*p* < 0.01) and TGFβ expression was also upregulated, although there was no significant difference (*p* > 0.05) when compared with the control group (Fig. 4b, 4c). In contrast to the RT and CT groups, positive scores arising from the IFNα treatment indicated no significant alteration in TGFβ and IL-10 expression levels (*p* > 0.05, Fig. 4).

## Discussion

In the treatment of melanoma, RT, CT, and interferon are all common adjuvant therapies after surgery. But while many in vitro and in vivo studies have shown that melanoma growth can be slowed with RT or CT therapy, little clinical success has been achieved. In addition, there is little known about the benefit of immunotherapeutic regimens either in basic studies or clinical research. This is important to determine as research has shown that different immune mechanisms appear to play a critical role in the biological behavior of melanoma. The purpose of this study was to examine the effect of different therapeutic regimens on the immune status and microenvironment of the tumors, in particular how the different treatments affected the levels of CD4^+^CD25^+^Foxp3^+^ Treg_s_, TGFβ, and IL-10.

The results of this study show that melanoma growth can be significantly inhibited by many of the examined therapies including RT, CT and IFNα. Despite the varying results of the tumor inhibition, the most effective was for the CT and RCT groups when compared with the RT and immunotherapy groups. From this we hypothesized that results are a function of the differing mechanisms underlying the treatments. The drug DTIC exerts its effect through indirect inhibition of cell metabolism and direct cytotoxicity. While RT has a limited ability to suppress melanoma growth, it may be used synergistically with CT to kill cancer cells. IFNα therapy is able to modulate the immune system, and through this, slow the growth of melanoma, although its ability to directly affect tumor growth is weak. We have observed the clinical efficacy of different treatments during practice, yet the effect of immunotherapy is relatively mild and it takes time for the treatment to take effect. We hypothesize that RT and CT generally enhance immunosuppression and decrease the immune response. The results of this work have revealed that a decrease of CD4^+^CD25^+^Foxp3^+^ Treg_s_ in the spleens of the mice after INFα treatment was most prominent compared with the levels found in mice treated with RT and CT. Also, a significant decrease in the CD4^+^CD25^+^Foxp3^+^ Treg_s_ levels in peripheral blood was detected in the INFα group, as revealed by flow cytometry (*p* < 0.05). These results suggest that immunotherapy with IFNα has the ability of down regulate CD4^+^CD25^+^Foxp3^+^ Treg_s_, yet the influence of RT and CT on CD4^+^CD25^+^Foxp3^+^ Treg_s_ levels is negligible.

It has recently been reported that IFNα can induce the MAPK/ERK (mitogen-activated protein kinases/extracellular signal-regulated kinases)-mediated phosphodiesterase four activation, and negatively affect cAMP in CD4^+^CD25^+^Foxp3^+^ Treg_s_, thus suppressing the function of Treg_s_ [18]. At the same time, the Jak-Stat1 (Janus Kinase- Signal transducers and activators of transcription 1) pathway can be stimulated by IFNα, which consequently activates effector T cells, natural killer (NK) cells, and dendritic cells, thereby indirectly enhancing the cytotoxic effect on tumor cells [15, 19–21]. Studies by Stergios et al. have suggested an indirect immunoregulatory mechanism of high-dose IFNα-2b which activates host immune cells to increase the cytocidal effect on cancer cells [21]. In this study, we have demonstrated that INFα is capable of significantly decreasing CD4^+^CD25^+^Foxp3^+^ Treg_s_ levels in the spleen and in peripheral blood, suggesting the immunomodulatory ability of INFα is to down regulate CD4^+^CD25^+^Foxp3^+^ Treg_s_. Many other studies have revealed that the ratio of CD4^+^CD25^+^Foxp3^+^ Treg_s_ to lymphocytes can be significantly upregulated in peripheral blood, in the spleen and in the lymphoids, and CD4^+^CD25^+^Foxp3^+^ Treg_s_s are resistant to γ-radiation [16, 22]. Our study has also showed the radio-resistance of CD4^+^CD25^+^ Foxp3^+^ Treg_s_ to a certain extent, particularly CD4^+^CD25^+^ Foxp3^+^ Treg_s_ in peripheral blood. Accordingly, we hypothesize that comprised lymphocytes exposed to irradiation and radio-resistant CD4^+^CD25^+^ Foxp3^+^ Treg_s_ that infiltrate into the microenvironment have been implicated in the immunosuppression of those patients given radiotherapy. Several other studies have suggested that the immune response of tumor cells can be modulated by fludarabine, paclitaxel and cyclophosphamide, by decreasing or depleting CD4^+^CD25^+^Foxp3^+^ Treg_s_ [23–25]. However, Tohyama et al. [17] have reported that chemotherapeutic agents, such as DTIC, have the ability to impair immunity by increasing CD4^+^CD25^+^Foxp3^+^ Treg_s_ and decreasing effector cells, which is consistent with our results. To this end, further studies are needed to investigate the clinical efficacy of different drugs on physical immunity.

Our study also used immunohistochemistry to detect the expression levels of TGFβ and IL-10 in the tumor microenvironment. The results showed that expression of TGFβ and IL-10 was significantly upregulated around tumor cells post RT, CT and RCT, indicating high immunosuppression in the tumor microenvironment. It has been reported that TGFβ and IL-10 are required for CD4^+^CD25^+^Foxp3^+^ Treg_s_ mediated immune suppression, which inactivates CD8^+^ T cells and NK cells [9, 14, 26, 27]. The combined results of this work and others indicates that increased levels of TGFβ and IL-10 post RT and CT, inhibit physical immunity as a result of RT and CT turning CD4^+^CD25^+^Foxp3^+^ Treg_s_ as predominant immune cells among lymphocyte population. From this, they suppress the recruitment of other effector cells, such as CTLs and NK cells, which therefore helps cancer cells to evade the immune system. In contrast to RT and CT, no significant alterations of TGFβ and IL-10 levels in the microenvironment were observed after IFNα-based immunotherapy.

## Conclusions

The present study showed that expression of Treg_s_ can be down regulated by IFNα, which reverses immunosuppression and makes the cancer cells more susceptible to treatment. The reason for why no significant down regulation of TGFβ and IL-10 was detected during IFNα treatment may due to short observing time, which also parallels the response processes of IFNα in clinical utilization. For differential therapeutic regimens have varying effects on physical immunity, the immune condition of patients post therapy should be considered when developing new treatment regimens.

## Abbreviations

APCs, antigen presenting cells; CCL22, chemokine (C-C Motif) Ligand 22; CT, chemotherapy; CTL, cytotoxic T cells; DC, dendritic cells; DTIC, dacarbazine; ERK, extracellular signal-regulated kinases; IL-10, interleukin-10; INF, interferon; Jak-Stat1 pathway, Janus Kinase- Signal transducers and activators of transcription one pathway; MAPK, mitogen-activated protein kinases; NK cells, natural killer cells; NS, normal saline; RCT, radiochemotherapy; RT, radiotherapy; TGFβ, transforming growth factor β; Treg_s_, regulatory T cells
